# Expression of Adiponectin Receptors on Peripheral Blood Leukocytes of Hypertensive Children Is Associated with the Severity of Hypertension

**DOI:** 10.1155/2015/742646

**Published:** 2015-06-04

**Authors:** Lidia Gackowska, Mieczyslaw Litwin, Joanna Trojanek, Andrzej Eljaszewicz, Izabela Kubiszewska, Anna Niemirska, Aldona Wierzbicka, Jacek Michalkiewicz

**Affiliations:** ^1^Chair of Immunology, Collegium Medicum in Bydgoszcz, Nicolaus Copernicus University of Torun, 85-094 Bydgoszcz, Poland; ^2^Department of Nephrology and Arterial Hypertension, The Children's Memorial Health Institute, 07-730 Warsaw, Poland; ^3^Department of Microbiology and Immunology, The Children's Memorial Health Institute, 07-730 Warsaw, Poland; ^4^Department of Biochemistry and Experimental Medicine, The Children's Memorial Health Institute, 07-730 Warsaw, Poland

## Abstract

The aim of the study was to find out whether peripheral blood leukocyte adiponectin receptors 1 and 2 (AdipoR1, AdipoR2) protein expression patterns (flow cytometry) differ between the primary hypertension children (*n* = 57) and healthy controls (*n* = 19) and if their expression levels are related to selected clinical parameters. The group of 26 patients [AdipoR(−)] showed lower and the group of 31 patients [AdipoR(+)] showed higher AdipoRs protein expression than the control and each other (*P* < 0.01 for neutrophils, *P* < 0.05 for monocytes). The AdipoR(+) leukocytes expressed higher AdipoR1 mRNA levels (RT-PCR) than AdipoR(−) ones and controls (*P* = 0.022 and *P* = 0.007, resp.). Despite greater BMI, the AdipoR(−) patients had unchanged serum adiponectin levels. In contrast, AdipoR(+) patients had lower serum adiponectin concentrations than the AdipoR(−) ones and controls (*P* < 0.001). The AdipoR(+) patients had higher blood pressure (*P* = 0.042) and greater carotid intima-media thickness (*P* = 0.017) than the AdipoR(−) ones. The stage of hypertension was associated with increased neutrophil but not monocyte AdipoR1 density (AdipoR1 MFI) (*P* < 0.05). Severe ambulatory hypertension was presented more often in AdipoR(+) patients than in AdipoR(−) ones (51.6% versus 26.9%, resp.; *P* < 0.01). In conclusion, neutrophil AdipoRs upregulation was associated with early stages of vascular injury, hypertension severity, and low serum levels of adiponectin.

## 1. Introduction

Primary hypertension (PH) is a complex disease consisting of hemodynamic, metabolic, and immune abnormalities. The primary reason for these defects is still unknown, but immune abnormalities are now considered to be the leading factors in the PH pathogenesis [1]. Low-grade systemic inflammation, including innate and adaptive immune response components, seems to play a role in the pathogenesis of PH [2]. It was found that the serum systemic inflammatory markers, including C-reactive protein, cytokines, and chemokines, are related mainly to metabolic abnormalities and oxidative stress components or even to target organ damage (TOD) but are rather weakly associated with blood pressure elevation per se [3]. Recently we found that peripheral blood leukocytes (PBLs) of PH children showed an upregulation of renin-angiotensin system (RAS) genes [4]. These observations, as well as other results, may indicate that leukocyte activation profiles are much more related to blood pressure elevation than the serum acute phase response components. The reason of this phenomenon is currently unclear, but its presence may suggest that human PH is related to some alterations in leukocyte functions, as it has previously been suggested in animal models [5]. We previously reported that the PH children serum adiponectin levels correlated negatively with increased carotid intima-media thickness [6, 7]. Here we hypothesized that the PH children PBLs adiponectin receptors (AdipoRs) expression profile might be related to a stage of arterial hypertension and TOD. We found here that upregulation of AdipoRs expression in the PH children leukocytes (neutrophils) was associated with both blood pressure elevation and TOD increase. This is a novel finding suggesting an engagement of the innate immune system in the development and maintenance of PH in children.

## 2. Patients and Methods

The study has been conducted according to the Declaration of Helsinki and with the approval of the Ethics Committee. All patients and parents gave consent to participate in the study. Out of 122 consecutively referred pediatric patients with newly diagnosed primary hypertension (PH), 57 children (43 boys), in mean age 15.0 ± 2.6 years, who underwent all laboratory procedures, were included in the study. The exclusion criteria were the presence of any significant chronic disease (except for PH), including diabetes and chronic kidney disease, any acute illness, including infections in the 6 weeks preceding enrollment, and incomplete data. Arterial hypertension was diagnosed according to the 4th Task Force Report and confirmed by 24-hour ambulatory blood pressure monitoring (ABPM) [8]. PH was diagnosed after exclusion of secondary hypertension according to guidelines. Blood pressure status was defined according to ABPM classification [9]. All hypertensive children underwent full protocol of assessment of TOD and biochemical investigations. The control group consisted of 19 (10 boys) healthy children, mean age 13.9 ± 3.5 years.

### 2.1. Anthropometric Measurements

Body mass index (BMI) and waist circumference (WC) were expressed as absolute values and as standard deviation scores (SDS), from the median of the norm. Current reference normative values of anthropometrical parameters for Polish population have been used to calculate SDS values [10].

### 2.2. Assessment of Target Organ Damage (TOD)

TOD was assessed using left ventricular mass index (LVMi), common carotid artery intima-media thickness (cIMT), and carotid wall cross-sectional area (WCSA).

### 2.3. Echocardiography

All echocardiographic (ECHO) examinations were performed by one examiner, who knew the clinical diagnosis but was unaware of the severity of hypertension and the effectiveness of treatment. ECHO measurements were performed according to the American Society of Echocardiography guidelines. To standardize the left ventricular mass to height, LVMi was calculated according to the de Simone formula [11]. Left ventricular hypertrophy (LVH) was defined as an LVMi value above the 95th percentile for age and gender, based on the reference data, and severe LVH as LVMi equal to or greater than 51 g/m^2.7^ [12].

### 2.4. IMT Measurements

cIMT was evaluated by ultrasound technique, according to the previously described methodology [13, 14]. Mean wall cross-sectional area of the carotid artery (WCSA) was calculated from the equation WCSA = *π*(dD/2 + IMT)2 − *π*(dD/2)2, where dD is the mean diastolic diameter and sD is the mean systolic diameter. Mean and standard deviation (SD) of normal values for cIMT and WCSA were taken from recently published normative data of cIMT for age- and sex-matched healthy children [13].

### 2.5. Laboratory Investigations

Blood samples were taken after 12 h of fasting. Lipid profile, serum uric acid (UA), and high sensitive C-reactive protein (hsCRP) were assessed in all patients. The hsCRP concentration was determined using highly sensitive immunoturbidimetry (Orion Diagnostica).

### 2.6. Immunoassays

Circulating levels of serum mediators were measured by means of commercially available ELISA kits: DuoSet, R&D Systems (MMP-9, TIMP-1, sCD14), DRG International Inc. (adiponectin, leptin), and BD Bioscience (IL-12p70, IL-1beta, and TNF-alpha), according to the manufacturer's instructions. Before performing the tests, the serum samples were diluted according to each kit's protocol and the final results were obtained by appropriate multiplication. The protein level in the diluted sample was calculated from a reference curve generated for a given assay by using reference standards containing known concentrations of appropriate protein. Results were expressed as nanograms or picograms per mL. Values below detection limit were considered as 0 [15, 16].

### 2.7. Assessment of Leukocyte Adiponectin Receptor Expression by Flow Cytometry

The leukocyte adiponectin receptor expressions (AdipoR1 and AdipoR2) were determined in heparin-collected whole blood samples with the use of direct and indirect three-color flow cytometry. First, the blood samples were washed with PBS, and 100 *μ*L of blood was incubated with primary, unstained antibodies: goat anti-human AdipoR1 and goat anti-human AdipoR2 (Santa Cruz Biotechnology Inc.) in two independent tubes. The blood samples were then washed with washing buffer, preincubated with donkey serum to avoid nonspecific binding, and stained with donkey anti-goat FITC-conjugated secondary antibodies (Santa Cruz Biotechnology Inc.). The erythrocytes were removed with the use of BD Bioscience FACS lysing solution. The stained leukocytes were suspended in cold PBS and analyzed immediately with flow cytometry (BD FACSCan). The cells stained solely with secondary antibody were used as negative control (isotype control) [17, 18]. For the leukocyte phenotype analysis, a gate was set on the neutrophil and monocyte by means of both the morphological features (a forward scatter FS versus 90° light scatter-side scatter SS) and the fluorescence characteristics of the cells labeled with phycoerythrin- (PE-) coupled anti-CD16 (neutrophils) and Peridinin Chlorophyll Protein Complex- (PerCP-) coupled anti-CD14 (monocytes) (BD Biosciences) as gating parameters. A histogram of log green fluorescence of each adiponectin receptor was used for the determination of the percentage of positive cells (%) and the mean fluorescence intensity (MFI) of each sample. The background fluorescence level for each specimen was established using the isotype control. The cells with AdipoRs fluorescence expression levels higher than those of the isotype controls were counted as positive: here as AdipoR(+). Accordingly, the cells with AdipoRs fluorescence expression levels lower than those of isotype controls were described here as AdipoRs(−). Autocomp using Calibrite beads (BD Biosciences) was used to standardize instrument electronics. Data acquisition was performed using the BD CellQuest Pro software (BD Biosciences) and the analysis with FlowJo 7.5.5 (Tree Star Inc.).

### 2.8. RNA Isolation and Real Time PCR Technique

PBLs were isolated from venous blood by means of Histopaque (Sigma-Aldrich 1119) gradient centrifugation. Total RNA was isolated with Chomczyński method using Trizol Reagent (Invitrogen), as described previously [4, 19]. RNA sample concentration was determined spectrophotometrically at 260 nm, and the purity was confirmed by 260/280 ratio. One microgram of total RNA for each sample was converted into cDNA in the Reverse Transcription Polymerase Chain Reaction (RT-PCR) by using Taq Man Reverse Transcription Reagents. Quantitative RT-PCR (real time PCR) for* ADIPOR1* target gene and endogenous control (reference gene) glyceraldehydes-3-phosphate dehydrogenase (G3PDH) were performed on Viia 7 Real Time System, according to the manufacturer's recommendation. For one reaction, 50 ng of cDNA with SYBR Green PCR Master Mix and 10 nmol/L of each of the forward and reverse primers [*ADIPOR1 *F: ACA AGG TCT GGG AGG GAC GT;* ADIPOR1 *R: CAT GGG AGG TCT ATG ACC ATG;* G3PDH* F: GCG GGG CTC TCC AGA ACA TCA T;* G3PDH* R: CCA GCC CCA GCG TCA AAG GTG] were applied, and analysis of each sample was performed at least in duplicate. The specificity of the amplification reaction was verified by analyzing the melting curve. The gene expression level was expressed as fold change of target gene expression according to basic level (=1) of the same gene expression in the control group's leukocytes, after normalization by expression of the reference gene G3PDH, by using Pfaffl's mathematical model, as previously described [4, 20]. All reagents, equipment, and other supplies for Q-PCR technique were provided by Applied Biosystems (now Life Technologies).

### 2.9. Statistical Analysis

Statistical analysis was performed using Statistica (StatSoft, Inc., 2011, STATISTICA, version 10.0, http://www.statsoft.com/). Normal distribution of the analyzed variables was tested with Kolmogorov-Smirnov test with Lilliefors correction. The *t*-test for independent measures and chi-square test were used for intergroup comparisons of continuous and categorical variables, respectively. Comparisons between groups presenting with different clinical stages were performed by using a generalized regression model (GRM), while linear regression analysis (Pearson's or Spearman's, depending on variable distribution) was performed to assess the relationship between continuous variables. In all analyses, *P* < 0.05 was considered as statistically significant. A probability value ranging between 0.05 and 0.1 was regarded as a statistical tendency. The results are depicted in the graphs in the form of mean values and their standard deviations.

## 3. Results

### 3.1. Assessment of Adiponectin Receptor Expression on Peripheral Blood Leukocytes

The group of 26 patients (45%) showed very low neutrophil and monocyte surface AdipoR1 and AdipoR2 expression levels (AdipoR(−)), both in terms of the percentage of positive cells and receptor density (MFI).

**Figure 1:**
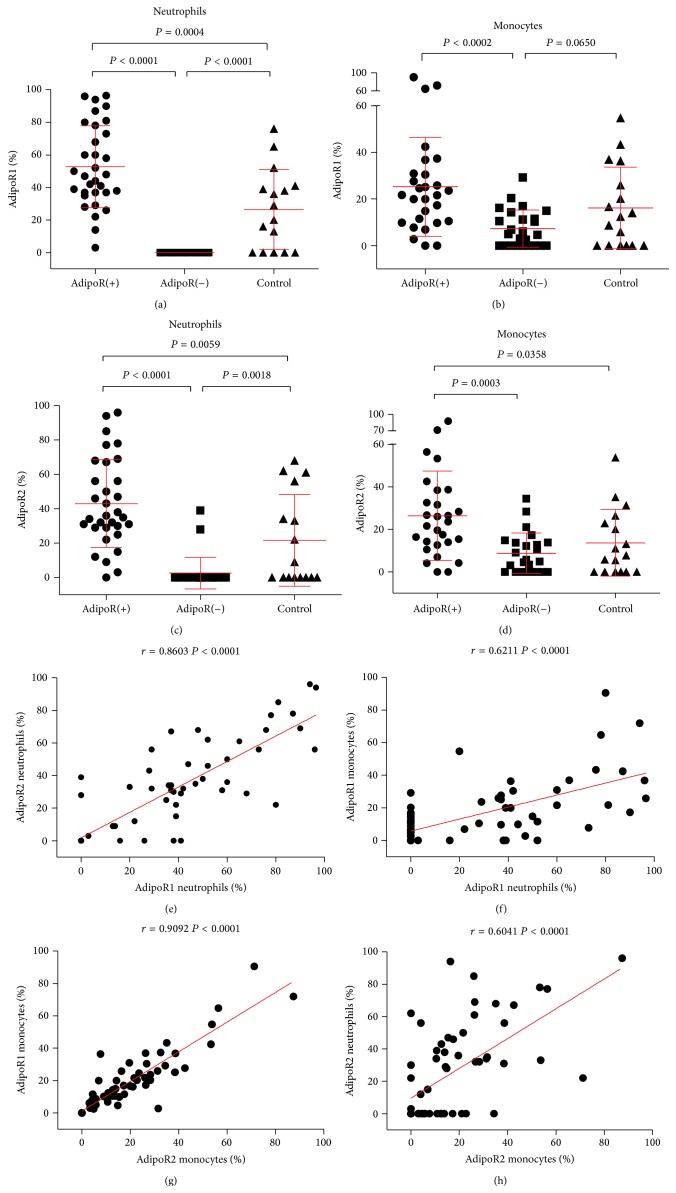
Expression and correlations of AdipoR on neutrophils and monocytes in PH children and normotensive control. The summary of analyses of AdipoR1 and AdipoR2 expression in neutrophils and monocytes in PH children (AdipoR(+) and AdipoR(−)) and normotensive control. (a) Percentage of neutrophils showing AdipoR1 receptor expression (AdipoR1%). (b) Mean levels of AdipoR1 receptor expression (AdipoR1%) in monocytes. (c) Percentage of neutrophils showing AdipoR2 receptor expression (AdipoR2%). (d) Mean levels of AdipoR2 receptor expression (AdipoR2%) in monocytes. All results (a–d) presented as means ± SD. AdipoR(+), *n* = 31, AdipoR(−), *n* = 26, controls, *n* = 19. (e) Correlations between AdipoR1 and AdipoR2 surface expression levels in neutrophils. (f) Correlations between the percentage of AdipoR1-positive neutrophils and AdipoR1-positive monocytes. (g) Correlations between the percentage of monocytes showing expression of AdipoR1 and AdipoR2. (h) Correlations between the percentage of AdipoR2-positive neutrophils and AdipoR2-positive monocytes.

In contrast, the other 31 patients (55%) showed a significantly greater proportion of AdipoR positive leukocytes (AdipoR(+)) than the AdipoR(−) ones and the controls (*P* < 0.01 for neutrophils, *P* < 0.05 for monocytes) (Figures 1(a)–1(d)). This distribution profile was dependent on neutrophils but not monocytes. As much as 29% and 48% of hypertensive children versus 16% and 21% of control had elevated proportion of AdipoR1 and AdipoR2 bearing neutrophils, respectively. In contrast, the percentage of patients having AdipoR1^high^ and AipoR2^high^ monocytes (25% and 30%, resp.) did not differ from that of control (26% and 21%, resp.).

The percentage of AdipoRs on neutrophils correlated with the proportion of AdipoR positive monocytes (*r* = 0.6211, *P* < 0.0001 for AdipoR1 and *r* = 0.6041, *P* < 0.0001 for AdipoR2). Both AdipoRs were correlated with each other (*r* = 0.8603, *P* < 0.001 for AdipoR1 versus AdipoR2 in neutrophils, and *r* = 0.909, *P* < 0.001 for AdipoR1 versus AdipoR2 in monocytes) (Figures 1(e)–1(h)).

Patients from AdipoR(−) and AdipoR(+) groups did not differ in terms of BMI and WC (Table 1). There were no statistical differences in the AdipoR1 and AdipoR2 leukocyte expression profiles between female and male patients (*P* > 0.05).

**Figure 2:**
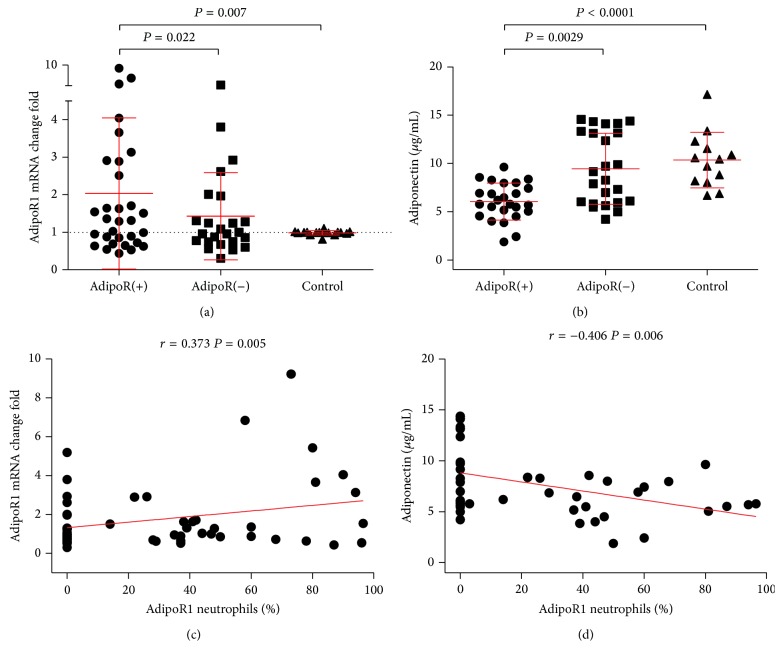
AdipoR1 mRNA expression in leukocytes and the serum adiponectin concentration in PH children and normotensive controls. (a) AdipoR1 gene expression levels (AdipoR1 mRNA). Results presented as means ± SD; AdipoR(+), *n* = 31, AdipoR(−), *n* = 16, controls, *n* = 24. (b) Serum adiponectin levels. Results presented as means ± SD; AdipoR(+), *n* = 25, AdipoR(−), *n* = 25, control, *n* = 13. (c) Correlations between the percentage of neutrophils showing expression of AdipoR1 and AdipoR1 mRNA leukocyte expression. (d) Correlations between the percentage of AdipoR1-positive neutrophils and serum adiponectin concentrations.

The leukocytes from AdipoR(+) patients showed significantly higher total leukocyte AdipoR1 mRNA levels than those from AdipoR(−) patients or from the controls (*P* = 0.022 and *P* = 0.007, resp.) (Figure 2(a)). AdipoR1 mRNA expression correlated positively solely with the percentage of AdipoR1-bearing neutrophils, but not monocytes (*r* = 0.373, *P* = 0.005) (Figure 2(c)).

### 3.2. Serum Parameters Distribution in the AdipoR(−) and AdipoR(+) Patients

AdipoR(+) patients had significantly lower adiponectin concentrations (6.0 ± 1.9 *µ*g/mL), as compared to AdipoR(−) patients (9.2 ± 3.8 *µ*g/mL) and controls (10.3 ± 2.9 *µ*g/mL) (*P* = 0.0029 and *P* < 0.001, resp.) (Figure 2(b)). Serum adiponectin levels correlated inversely with AdipoR-bearing neutrophil, but not monocyte or leukocyte AdipoR1 mRNA expression (*r* = −0.406, *P* = 0.006) (Figure 2(d)).

AdipoR(+) patients had slightly, albeit significantly, lower sCD14 concentration (779.4 ± 174.1 ng/mL) than the AdipoR(−) ones (885.4 ± 304.2 ng/mL) and controls (998.5 ± 152.3 ng/mL) (*P* < 0.01, Table 1). sCD14 levels were inversely related to neutrophil and monocyte AdipoR protein expressions (Table 2) and tended to correlate positively with adiponectin levels (*r* = 0.318, *P* = 0.08).

The serum concentrations of leptin, sCRP, MMP-9, TIMP1, IL-12p70, and IL-1 beta did not correlate with leukocyte AdipoR expression profile, on either protein or mRNA levels (data not shown).

### 3.3. Relation of Clinical Variables and Target Organ Damage Markers to Leukocyte AdipoR Expression Profiles

**Figure 3:**
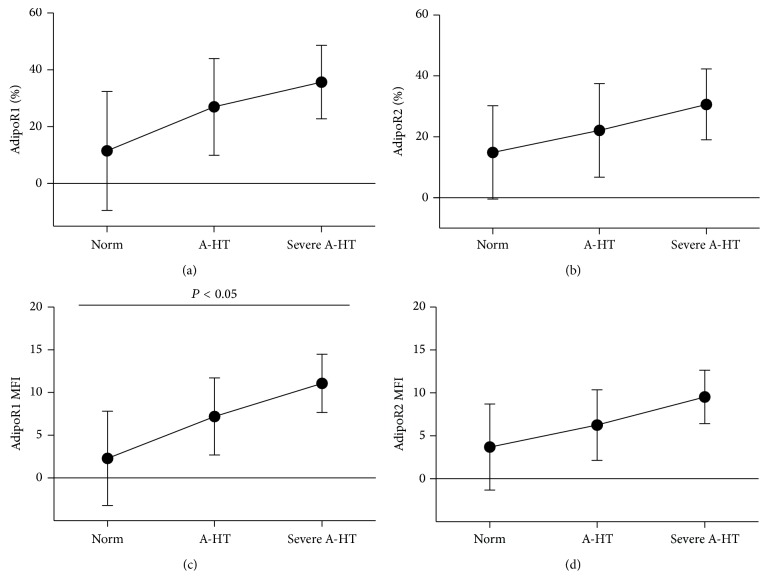
Expression of adiponectin receptors in neutrophils and stage of hypertension. Results presented as means ± SD. A-HT, ambulatory hypertension; (a) AdipoR1% and (b) AdipoR2%, percentage of neutrophils showing expression of adiponectin receptors; (c) AdipoR1 MFI and (d) AdipoR2 MFI, mean density of adiponectin receptors on the surface of neutrophils expressed as mean fluorescence intensity of positive cells.

The AdipoR(+) patients had significantly higher mean 24-hour systolic blood pressure and higher cIMT values than the AdipoR(−) patients (Table 1). Furthermore, neutrophil AdipoR1 mean fluorescence intensity (MFI) values positively correlated with the 24-hour SBP values (*r* = 0.26, *P* = 0.045), and the proportions of AdipoR2 bearing neutrophils were related to 24-hour DBP (*r* = 0.28, *P* = 0.043). Disease severity, as assessed by the stage of hypertension, was related to neutrophil but not monocyte AdipoR upregulation. The stage of hypertension (according to ABPM blood pressure classification) was associated with increased neutrophil AdipoR1 fluorescence intensity (AdipoR1 MFI) (*P* < 0.05) (Figure 3(c)).

Severe ambulatory hypertension also presented more often in patients with high AdipoR expression, as compared to the AdipoR(−) ones (51.6% versus 26.9%, resp.; *P* < 0.01). The neutrophil AdipoR1 and AdipoR2 protein expression profiles were not associated with anthropometric data, such as the age, body height, body weight, or BMI. In striking contrast, the monocyte AdipoR protein expression (MFI), as well as total leukocyte AdipoR1 mRNA, was both inversely related to the anthropometric parameters (Table 2), as well as to adiponectin levels (*r* = −0.297, *r* = −0.484, *r* = −0.445, and *r* = −0.335, resp., *P* < 0.05). Furthermore, LVMI did not differ between the patient groups (Table 1) but also correlated negatively with the monocyte but not neutrophil, AdipoR MFI (Table 2).

## 4. Discussion

The analysis of PBL gene expression pattern proved to be useful to describe intermediate phenotype of the hypertensive disorder. There is a high gene expression concordance rate (over 80%) between PBLs and other tissues, both in humans and other species [21]. Leukocytes continuously interact with virtually every tissue and organ in the body and thus their gene and surface receptor expression profiles may serve as early markers of many abnormalities [22]. Moreover, leukocytes are important mediators of TOD in animals with experimentally induced hypertension [1, 23, 24].

The AdipoR1 and AdipoR2 protein expression levels were the highest in monocytes and neutrophils, but they were relatively low in lymphocytes (not shown). These data are consistent with the results published by Pang and Narendran [18] and Rossi and Lord [25]. We found that neutrophil and monocyte AdipoRs protein expression levels were closely related to each other and to total leukocyte AdipoR1 mRNA. However, the PBLs of PH children showed either high (AdipoRs(+) patients) or low AdipoR expression (AdipoR(−) patients) profiles, which were strictly related to different disease severity and TOD. Only AdipoR(+) patients presented with increased cIMT and had severe ambulatory hypertension, alongside low serum adiponectin concentrations. Strikingly, neutrophil (but not monocyte) AdipoR protein expression levels (MFI) were negatively related to serum adiponectin concentrations but positively related to the severity of hypertension. These changes occurred independently of other studied systemic factors, including biochemical, anthropometric, and immune parameters.

In contrast to neutrophil, monocyte AdipoR expression levels (expressed as MFI), as well as total AdipoR1 mRNA, turned out to be independent of serum adiponectin concentrations but showed negative correlations with some serum immune mediators (sCD14, TNF-alpha), anthropometric parameters (body mass, BMI, WC), and LMVI (Table 2).

Altogether, these data suggest that neutrophil AdipoR upregulation in conditions of low adiponectin levels is related to vascular changes (described hereby as increased cIMT) and disease severity. The involvement of neutrophils in the pathogenesis of PH and vascular changes has long been postulated. Hypertensive adult patients exhibit several alterations in neutrophil characteristics, including an increased neutrophil count [26] and activated neutrophil functions, such as upregulation of CD11b and rapid release of neutrophil primary granules (CD69 expression) on short incubation with PBS or TPA [27]. Similarly, PBLs from PH children have been revealed to show an altered expression of inflammatory molecules and elements of renin-angiotensin system. This altered pattern of gene expression changed during nonpharmacological treatment [4].

Our study showed that different neutrophil AdipoR expression profiles were associated with hypertension severity and subclinical arterial injury. Neutrophils normally show AdipoR expression levels that are equal to those of monocytes, as found also in our present study, and both of them react with adiponectin [25]. Adiponectin has been shown to modulate both neutrophil phenotype and functions, but its significance in the PH development remains unknown. Adiponectin exerts a variety of effects related to neutrophil biology, including (a) inhibition of* E. coli* phagocytosis by suppression of PKB and ERK1/2 MAPK signaling pathways, and Mac-1 activation [25], (b) suppression of reactive oxygen species formation by inhibition of NADPH oxidase activation [28], (c) increase in neutrophil survival rate by inhibition of their apoptosis via activation of AMPK, PI3K/PKB, and ERK pathways, stabilization of antiapoptotic Mc1-1 molecule, and reduction of cell membrane ceramide accumulation [29], (d) downregulation of granulopoiesis by suppression of GM colony formation [30], and (e) acting as growth factor for hematopoietic stem cells [31].

In the steady state, serum adiponectin levels are rather low, but its receptor expression levels are higher in PBLs of obese people than in the control subjects, possibly due to alterations in insulin levels and insulin sensitivity [32]. Adiponectin increases insulin sensitivity, but insulin downregulates both AdipoR expression (especially AdipoR1) and insulin sensitivity [33]. In conditions of insulin deficiency (like in type 1 diabetes) AdipoR expression is high, but serum adiponectin levels are also elevated [34]. Furthermore, increased serum adiponectin level has been associated with higher all-cause mortality in type 1 diabetes patients [35]. On the other hand, reduced or low serum adiponectin levels have long been linked to metabolic syndrome components, such as visceral obesity and insulin resistance [36], and PH children with low serum adiponectin concentrations had increased cIMT [6, 7]. Our study showed that greater expression of AdipoRs on PBLs was associated with low serum adiponectin levels, blood pressure elevation, and increased cIMT. However, it is difficult to distinguish if increased cIMT results from lower adiponectin concentrations or higher blood pressure. Our findings are consistent with the previous reports indicating that hypoadiponectinemia is a strong predictor of future hypertension, even after adjusting for the confounding systemic factors, such as mean blood pressure, C-reactive protein, BMI, and WC [37]. In fact, these factors were not associated with neutrophil AdipoR upregulations in our patients. This suggests that other, probably genetic, factors control both neutrophil AdipoR expression profile and adiponectin levels.

In contrast to neutrophils, AdipoR expression on monocytes did not correlate with hypertension severity, expressed here as cIMT values and blood pressure elevation. Furthermore, we found inverse correlation between monocyte AdipoR expression level and LVMI. This finding corresponds with the recent report on the role of neutrophils, but not monocytes, in the left ventricular hypertrophy caused by angiotensin II infusion in mice [23].

Monocyte AdipoR expression profiles have been shown to be related to coronary artery disease (CAD) progression in adult patients, in whom AdipoR mRNA and protein upregulation correlated with arterial stiffness and cIMT. However, contrary to our results, AdipoR upregulation was positively related to systemic low-grade inflammation markers (increase in serum MMP-9 and CRP levels) and elevation of serum adiponectin concentration [22, 38, 39]. Furthermore, serum adiponectin levels, monocyte AdipoR expression, and adiponectin-induced synthesis of IL-10 by monocytes were downregulated in the overweight CAD patients, as compared to overweight patients without CAD [39]. Altogether, these data point to a protective role of AdipoR monocyte expression in CAD patients.

In contrast to the abovementioned adults with clinically evident cardiovascular disease, the PH children present the earliest stage of the pathological process. Thus, increased neutrophil, but not monocyte, AdipoR expression that was related to low serum adiponectin, may reflect the first phase of arterial disease (increased cIMT and blood pressure) that occurs without other systemic inflammatory effects. Moreover, PH is not the same as atherosclerosis and CAD which are distinct clinical entities although they may share common risk factors with PH. Some discrepancies may also be explained in terms of the technical differences. We tested AdipoR expression in whole blood leukocytes with flow cytometry (in the cells gated as monocytes and neutrophils) and with RT-PCR (total leukocyte AdipoR1 mRNA). In contrast, the authors mentioned above used PBMC (flow cytometry) and monocyte AdipoR1/2 mRNA. The isolation procedure may change both distribution of leukocyte subpopulations and their activation state, thus altering the profile of AdipoR expression.

We found a slight, albeit significant, increase in serum sCD14 levels (in the AdipoR(−) patients) that were inversely related to neutrophil and monocyte AdipoR expression and tended to correlate positively with serum adiponectin concentrations. These findings may suggest that lipopolysaccharides (LPS) (often present in obese individuals) of the AdipoR(−) patients can be bound by sCD14, transferred to HDL serum fraction and subsequently inactivated. Thus, CD14- and toll-like receptor-dependent inflammatory reactions are limited [40–42]. This effect may eventually be facilitated by adiponectin [43] and sCD14 can also limit the amount of LPS bound by monocytes, by increasing LPS efflux from these cells [44]. On the other hand, sCD14 elevation was reported to be associated with an increase in arterial stiffness in adult hypertensive patients [45]. Considering pro- or anti-inflammatory actions of sCD14 proteins, their significance in AdipoR- and adiponectin-controlled immune responses [41, 46] warrants further investigations.

## 5. Conclusion

To the best of our knowledge, there are no other data concerning the leukocyte AdipoR expression profiles in the onset of PH in children. We found that upregulation of neutrophil AdipoR expression was associated with early stages of vascular injury, hypertension severity, and low serum levels of adiponectin. These phenomena were not associated with involvement of systemic activities concerning immune, metabolic, and anthropometric parameters. Inverse associations between AdipoR expression profiles on neutrophils and monocytes and serum adiponectin concentrations are worth emphasizing. Our findings point to different roles of neutrophils and monocytes in the pathogenesis of PH and arterial injury; earlier activation of neutrophils may result in early vascular inflammation and subsequent monocyte involvement that is related to more systemic responses.

The main limitation of the study is its cross-sectional design. It does not allow assessing changes in AdipoR expression related to treatment. Similarly, it does not allow evaluating if treatment efficacy is associated with AdipoR expression. The other limitation is associated with measuring total adiponectin concentrations, rather than its different molecular forms. The strength of the study is recruitment of homogenous population of yet untreated hypertensive children without other cardiovascular risk factors, such as diabetes, smoking, and widespread atherosclerosis. Moreover, all children underwent full diagnostic approach with the assessment of hypertensive TOD. It allows the analysis of immune phenomena on the earliest phase of cardiovascular disease.

The results of our study give perspectives for further analysis of leukocyte-mediated, AdipoR-dependent immune reactions, and their role in the development of PH and hypertensive TOD. The relationship between insulin resistance and leukocyte AdipoR expression profiles still remains unclear. Also the effects of both nonpharmacological and pharmacological treatment on leukocyte AdipoR expression pattern were not studied. One may assume that different antihypertensive drugs may exert different effects on AdipoR expression profiles. Furthermore, the patients who differ in terms of AdipoR expression profiles may also differ in their responsiveness to pharmacological or nonpharmacological treatment. The other challenge is the analysis of different roles of neutrophils and monocytes in the pathogenesis of hypertensive TOD, especially in relation to absence or presence of AdipoRs in connection to other leukocyte phenotype characteristics, such as expression of ROS and apoptosis markers. The other issue is description of AdipoR patterns in relation to sex and ethnicity of PH children.

## Figures and Tables

**Table 1:** Comparison of demographic, anthropometric, and laboratory data in control group and AdipoR(+) and AdipoR(−) group of hypertensive children.

Variables	Control (*n* = 19)	Group of hypertensive children		
		AdipoR(+) (*n* = 31)	AdipoR(−) (*n* = 26)	*P* Value AdipoR(+) *versus* AdipoR(−)
Age (years)	13.9 ± 3.5	15.0 ± 2.6	15.0 ± 2.6	0.964
Growth, cm	157.8 ± 22.9	170.7 ± 14.8^†^	167 ± 13.8	0.383
Weight, kg	55.1 ± 21.8	75.5 ± 17.9^†^	72.5 ± 20.5^‡^	0.570
BMI	21.0 ± 4.3	25.6 ± 4.3^†^	25.5 ± 4.6^‡^	0.896
BMI-SDS	0.5 ± 1.0	1.3 ± 0.8^†^	1.3 ± 0.9^‡^	0.942
Waist circumference, cm	68.8 ± 8.5	82.7 ± 8.3^†^	83.6 ± 12.9^‡^	0.782
Waist circumference SDS	0.7 ± 0.6	1.4 ± 0.7	1.3 ± 1.1	0.690
24 h SBP, mm Hg	110.3 ± 14.4	130.5 ± 8.7^†^	125 ± 9.4^‡^	0.042^*^
24 h DBP, mm Hg	62.7 ± 5.5	73.0 ± 5.7^†^	71.1 ± 6.1^‡^	0.225
24 h MAP, mm Hg	88.6 ± 9.8	92.3 ± 5.9	89.4 ± 5.9	0.073
24 h heart rate, beats/min	75.1 ± 13.8	78.0 ± 14.1	79.1 ± 13.4	0.740
Left ventricular mass index, g/m^2.7^	28.2 ± 5.8	32.7 ± 6.5	34.5 ± 8.9	0.383
Carotid intima-media thickness, m	0.42 ± 0.03	0.45 ± 0.04	0.42 ± 0.04	0.017^*^
Carotid intima-media thickness *Z*-score	0.7 ± 0.7	1.57 ± 1.2	1.04 ± 1.0	0.073
Carotid wall cross-sectional area, mm^2^	6.8 ± 0.9	8.0 ± 1.5^†^	7.5 ± 1.4	0.092
Total cholesterol, mg/DL	178.6 ± 28.3	171.8 ± 35.1	174.5 ± 30.3	0.760
HDL cholesterol, mg/dL	53.4 ± 19.8	50.9 ± 13.9	51.4 ± 13.8	0.890
LDL cholesterol, mg/dL	109.0 ± 24.7	96.7 ± 29.4	101.9 ± 25.0	0.471
Triglycerides, mg/dL	85.9 ± 42.0	127.2 ± 79.3	111.7 ± 50.5	0.389
Uric acid mg/dL	5.1 ± 0.9	6.0 ± 1.4	5.8 ± 1.2	0.457
hsCRP, mg/L	0.2 ± 0.08	0.32 ± 0.2	0.28 ± 0.1	0.488
sCD14 ng/mL	998.5 ± 152.3	779.4 ± 174.1^†^	885.4 ± 304.1	0.008^*^

Results presented as mean ± SD. BMI indicates body mass index; DBP, diastolic blood pressure; HDL, high-density lipoprotein, hsCRP, high sensitivity C-reactive protein; LDL, low-density lipoprotein; MAP, mean arterial pressure; SBP, systolic blood pressure. Statistically significant differences are given as follows: ^*^AdipoR(+) *versus* AdipoR(−), ^†^AdipoR(+) *versus* control, ^‡^AdipoR(−) *versus* control, *P* < 0.05 is considered significant.

**Table 2:** Correlation of adiponectin receptors and AdipoR1 mRNA with selected cytokines and anthropometric parameters and LVMI.

	Neutrophils				Monocytes				
	AdipoR1		AdipoR2		AdipoR1		AdipoR2		AdipoR1 mRNA
	*r* [%]	*r* [MFI]	*r* [%]	*r* [MFI]	*r* [%]	*r* [MFI]	*r* [%]	*r* [MFI]	
sCD14	−0.257^*^	−0.307^*^	−0.339^*^	−0.384^*^	−0.289^*^	−0.342^*^	−0.291^*^	−0.429^*^	−0.050
Body mass	0.012	0.032	0.069	0.023	−0.043	−0.300^*^	−0.044	−0.333^*^	−0.298^*^
BMI	0.019	0.020	0.009	−0.030	0.075	−0.349^*^	0.017	−0.371^*^	−0.079
WC	−0.095	−0.063	−0.099	−0.106	−0.017	−0.336^*^	−0.092	−0.365^*^	−0.370^*^
LVMI	−0.023	−0.039	−0.116	−0.065	−0.197	−0.314^*^	−0.189	−0.331^*^	−0.067

AdipoR indicates adiponectin receptor, BMI, body mass index; WC, waist circumference, and LVMI, left ventricular hypertrophy. ^*^ *P* < 0.05 is considered significant.

## Abbreviations

TOD: Target organ damage
PBLs: Peripheral blood leukocytes.
